# Chromosome Conformation Capture Reveals Two Elements That Interact with the *PTBP3* (*ROD1*) Transcription Start Site

**DOI:** 10.3390/ijms20020242

**Published:** 2019-01-09

**Authors:** Marta Kubiak, Anna Jurek, Katarzyna Kamińska, Janusz Kowalewski, Sui Huang, Marzena Anna Lewandowska

**Affiliations:** 1 Innovative Medical Forum, The F. Lukaszczyk Oncology Center, Department of Molecular Oncology and Genetics, 85-796 Bydgoszcz, Poland; kubiak.marta@co.bydgoszcz.pl (M.K.); ania.m.jurek@gmail.com (A.J.); kaminskak@co.bydgoszcz.pl (K.K.); 2 The Ludwik Rydygier Collegium Medicum, Department of Thoracic Surgery And Tumours, Nicolaus Copernicus University, 85-796 Bydgoszcz, Poland; kowalewskij@co.bydgoszcz.pl; 3 Department of Cell and Molecular Biology, Northwestern University Feinberg School of Medicine, Chicago, IL 60611, USA; s-huang2@northwestern.edu

**Keywords:** chromatin conformation, PTBP3, ROD1 (Regulator Of Differentiation 1), cancer epigenetic, gene expression regulation, prostate cancer

## Abstract

The long-range control of gene expression is facilitated by chromatin looping and can be detected using chromosome conformation capture—3C. Here we focus on the chromatin architecture of the *PTBP3* (Polypyrimidine tract binding protein 3) locus to evaluate its potential role in regulating expression of the gene. *PTBP3* expression in prostate cancer cell lines is found significantly higher compared to skin fibroblasts using real-time PCR (*p* < 0.05) and digital droplet PCR (*p* < 0.01). Exploration of the chromatin spatial architecture of a nearly 200-kb fragment of chromosome 9 encompassing the *PTBP3* gene identified two elements located 63 kb upstream and 48 kb downstream of *PTBP3*, which looped specifically to the *PTBP3* promoter. These elements contain histone acetylation patterns characteristic of open chromatin regions with active enhancers. Our results reveal for the first time that long-range chromatin interactions between the −63 kb and +48 kb loci and the *PTBP3* promoter regulate the expression of this gene in prostate cancer cells. These interactions support an open chromatin form for the *PTBP3* locus in cancer cells and the three-dimensional structural model proposed in this paper.

## 1. Introduction

Regulation of gene expression involves multiple mechanisms at various stages leading to decreased or increased protein synthesis. Gene regulation at the transcription level is a combination of effects of chromatin conformation and interaction between the complex transcription machinery and DNA [1]. Post-transcriptional modifications control gene expression at the mRNA stage [2], and translational regulation affects the level of protein synthesized from mRNA by, for instance, controlling ribosome recruitment [3]. Epigenetic mechanisms involve DNA modification or post-translational modification of proteins that are closely associated with DNA as key mediators [4].

Some of the best known gene regulation mechanisms which play an important role in cancer development are: DNA methylation, gene silencing by microRNA or histone modifications [5]. In the recent years, chromatin was elevated to a key position as a mediator of the transformation of a normal cell into a malignant state [6]. Modifications such as acetylation of histones (H3K27ac, H3K9ac) and their methylation (H3K4, H3K36) may activate spatial organization of chromatin structure to produce a transcriptionally activated state [7]. Regulatory elements may interact with target genes or other regulatory elements over large genomic distances or can even activate the expression of genes located on different chromosomes [8]. Over the last 15 years, the development of Chromosome Conformation Capture (3C) and its subsequent high-throughput variants (4C [9,10], 5C [11], HiC [12], T2C [13], Capture C [14]) has given insights to better understand chromatin 3D organization and its correlation with the regulation mechanisms of gene expression [15].

*PTBP1* (Polypyrimidine tract binding protein 1,l hnRNP I) is a gene encoding a multifunctional protein mainly known for its role in tissue-specific regulation of exon splicing [16,17,18]. *PTBP1* is a member of a larger family of 4 genes in mammals: *PTBP1*, *PTBP2*, *PTBP3* and *smPTB*, characterized by a similar protein architecture and containing four RNA-recognition motifs (RRMs) [19]. We were interested in a *PTBP1* paralog, the *PTBP3(ROD1)* gene (Polypyrimidine tract binding protein 3, NM_005156), which plays a role in the negative regulation of differentiation [20]. *ROD1* is expressed preferentially in hematopoietic cells and has a known role in nonsense-mediated mRNA decay (NMD) [21]. Cross-regulation between *PTBP1* and *PTBP3* by non-productive alternative splicing has been reported [22], but other regulation mechanisms remain to be clarified. The *PTBP3* regulation mechanisms have not been thoroughly characterized.

Chromatin loops promote activation of gene expression at the mRNA level. Activation of gene transcription by three-dimensional chromatin architecture has been demonstrated in several genes, e.g., *CFTR* (Cystic fibrosis transmembrane conductance regulator) [23,24,25], *TNFAIP3* (NF Alpha Induced Protein 3) [26], *PPARγ2* Peroxisome Proliferator Activated Receptor Gamma 2) [27]. We observed a significant change in the *PTBP3* mRNA levels in cancer cell lines versus normal cells, consistent with high expression in lung cancer [28] and stomach cancer [29]. Therefore, we attempted to use the 3C technique to investigate whether the chromatin architecture of the *PTBP3* gene regulates its expression at the transcriptional level in cancer cells.

## 2. Results

### 2.1. Levels of PTBP3 mRNA in Prostate Cancer Cell Lines

**Figure 1:**
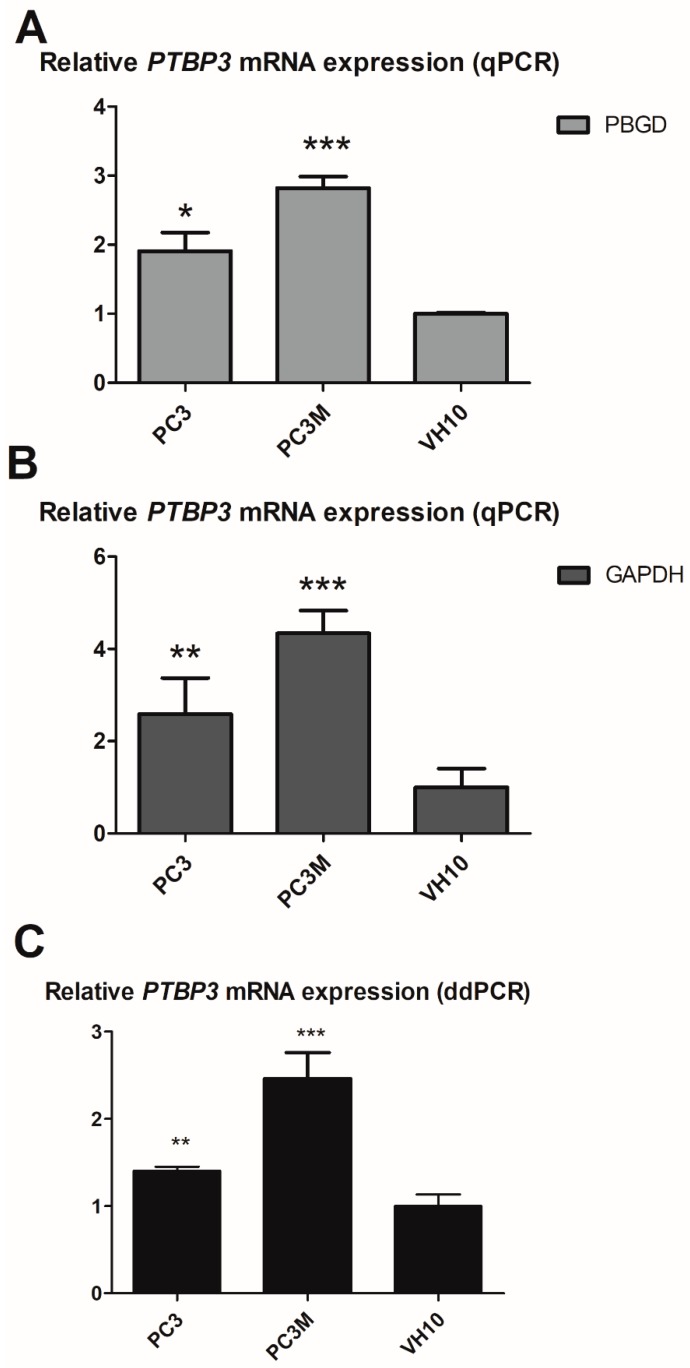
Comparison of the expression of *PTBP3* in prostate cancer cell lines. Asterisks represent statistically significant changes in the *PTBP3* mRNA levels in prostate cancer cells versus human fibroblasts * *p* < 0.5; ** *p* < 0.01; *** *p* < 0.001. Relative levels of *PTBP3* mRNA using quantitative PCR (qPCR) assay. Gray columns represent experiment performed in triplicates with references genes: (**A**) *PBGD* and (**B**) *GAPDH*; (**C**) *PTBP3* mRNA level using ddPCR assay. Stars represent statistically significant changes in the *PTBP3* mRNA levels versus human fibroblasts. Black columns represent experiments performed at least in triplicates.

To determine whether the mRNA levels of *PTBP3* differed between cancerous and non-cancerous cells, two quantitative PCR methods were used: Quantitative reverse transcriptase real-time PCR (qRT-PCR) and digital droplet PCR (ddPCR). The qRT-PCR assay using the Universal ProbeLibrary (UPL) probes involved mRNA level analysis normalized using glyceraldehyde-3-phosphate dehydrogenase (*GAPDH*) and porphobilinogen deaminase (*PBGD*). The results were consistent for both housekeeping genes (Figure 1). *PTBP3* was increased in prostate cancer cell lines compared to skin fibroblasts with *GAPDH* as the reference gene. The most significant increase in the *PTBP3* mRNA level was observed in the prostate cancer cell lines: PC3M cells (+/− 4.5-fold, *p* < 0.001) and the PC3 cells (+/− 2-fold, *p* < 0.01) compared to skin fibroblasts. With *PBGD* as the reference gene, the most significantly increased level of *PTBP3* mRNA was again observed in the PC3M cells (+/− 3-fold, *p* < 0.001) and the PC3 cells (+/− 2-fold, *p* < 0.05) vs. skin fibroblasts (Figure 1). As in the qPCR assay, in digital droplet PCR (ddPCR) the most significant increase in the *PTBP3* expression was observed in the PC3M cells (+/− 2.5-fold, *p* < 0.001) and the PC3 cells (+/− 1.4-fold, *p* < 0.01) compared to skin fibroblasts (Figure 1).

### 2.2. Looping Organization of the PTBP3 Locus in Prostate Cancer Cell Lines

To investigate the mechanism of higher level of *PTBP3* in cancer cells, we examined chromatin interaction of the *PTBP3* locus. We used chromosome conformation capture (3C) for the evaluation of long-range chromatin interaction across distant regulatory elements and active *PTBP3* promoter. We performed a 3C experiment using prostate cancer cell lines (PC3 and PC3M) with a statistically significant high expression of *PTBP3* versus human skin fibroblasts to identify chromatin interaction across the *PTBP3* locus.

**Figure 2:**
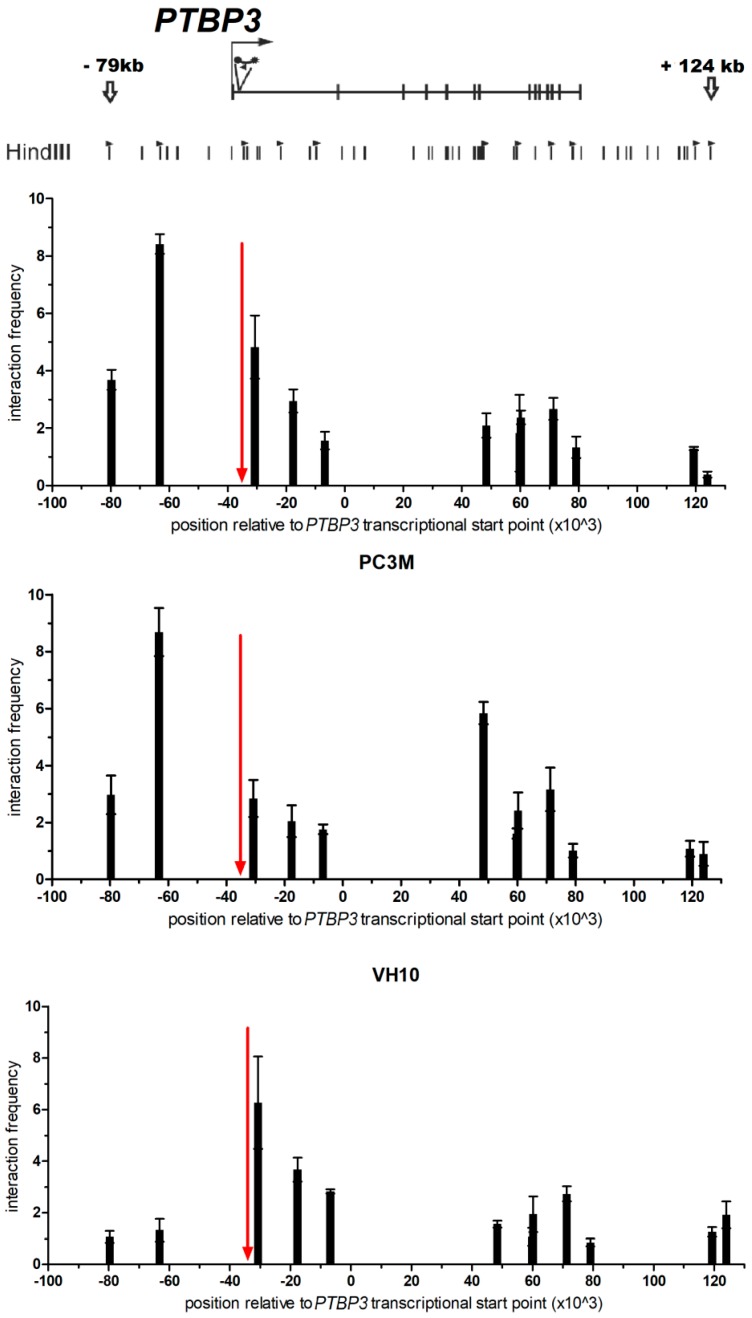
Two elements interacting with the *PTBP3* transcription start site (TSS) revealed by chromosome conformation capture (3C). Schematic representation of the PTBP3 gene with exons marked with vertical bars and the TSS represented by a bent arrow. Small vertical lines under the gene indicate HindIII restriction sites, and arrow heads indicate the localization of 3C primers (black arrows—forward; white arrow—reverse). The black circle with a star represent Taqman probe (bait). The X axis represents the position relative to the TSS. The Y axis represents the interaction frequency relative to the interaction frequency between two HindIII fragments within the ubiquitously expressed ERCC3 (ERCC Excision Repair 3) gene. The red arrow represents the bait region of the PTBP3 promoter, which includes a primer and a Taqman probe. The 3C experiment was performed in triplicates for each 3C library.

To analyze three-dimensional chromatin organization of the *PTBP3*, we used a 5′-3′ROD1 Reverseprimer, located in the 5′ region upstream of the promoter of *PTBP3*, and a set of forward primers across a 200-kb region encompassing the *PTBP3* locus and flanking regions (Figure 2, Supplementary Table S1). Within the primary human skin fibroblasts, which have a low level of expression of the *PTBP3* gene, interaction frequency with the *PTBP3* promoter decreased as a function of distance from the promoter, with no significant interaction between the promoter and either of the distal fragments located across the *PTBP3* gene.

In prostate cancer cell lines PC3 and PC3M, which express high levels of *PTBP3* transcripts, 3C analysis demonstrated chromatin interaction between the *PTBP3* promoter and distal or proximal fragments encompassing regulatory elements.

The highest chromatin interactions within the *PTBP3* promoter and distal regions of the gene were observed in the PC3M cell lines with the highest *PTBP3* expression. Frequent chromatin interactions were observed for two restriction fragments upstream and downstream of the *PTBP3* promoter. One region, encompassing 63 kb upstream of the TSS, was a fragment also observed in the PC3 cells with a 3-fold higher interaction than that observed in a restriction fragment located close to the promoter, and 9-fold higher than the background of non-specific interactions. Another fragment with a high chromatin interaction was identified at the 48 kb region downstream of the transcription start point. This interaction was 3-fold higher than that observed in a fragment located close to the *PTBP3* promoter and 6-fold higher than the background (Figure 2). The interacting fragments located 63 kb upstream and 48 kb downstream from the promoter region demonstrated a greater interaction frequency with the *PTBP3* promoter than a HindIII fragment located close (<4 kb) to the *PTBP3* promoter (Figure 2).

In the PC3 cells with a significant overexpression of *PTBP3*, frequent chromatin interactions were observed between the promoter fragment and its nearby restriction fragments. Additionally, frequent interactions with another restriction fragment were found, as evidenced by a local peak in interaction frequency. The interacting fragment was located in the flanking region of the 5′ end of *PTBP3* encompassing 63 kb upstream of the TSS. The chromatin interaction between the promoter and the fragment at −63 kb was approx. 2-fold higher than that between a fragment located close to the promoter, and approx. 9-fold higher than the background.

### 2.3. The −63 kbp and +48 kbp Regions of the PTBP3 Promoter are Open Chromatin Regions

To verify open chromatin state at the −63 kb and +48 kb locations of *PTBP3* in the PC3 and PC3M cell lines, chromatin immunoprecipitation (ChIP) with antibodies specific for acetylation of histone H3 on lysine 27 (H3K27Ac) that marked both active promoters and distal enhancers was conducted [30,31] Primer sets for the SYBR green qPCR were designed to amplify the regions of interests (−63 kb and +48 kb) upstream and downstream of the *PTBP3* promoter region, respectively.

**Figure 3:**
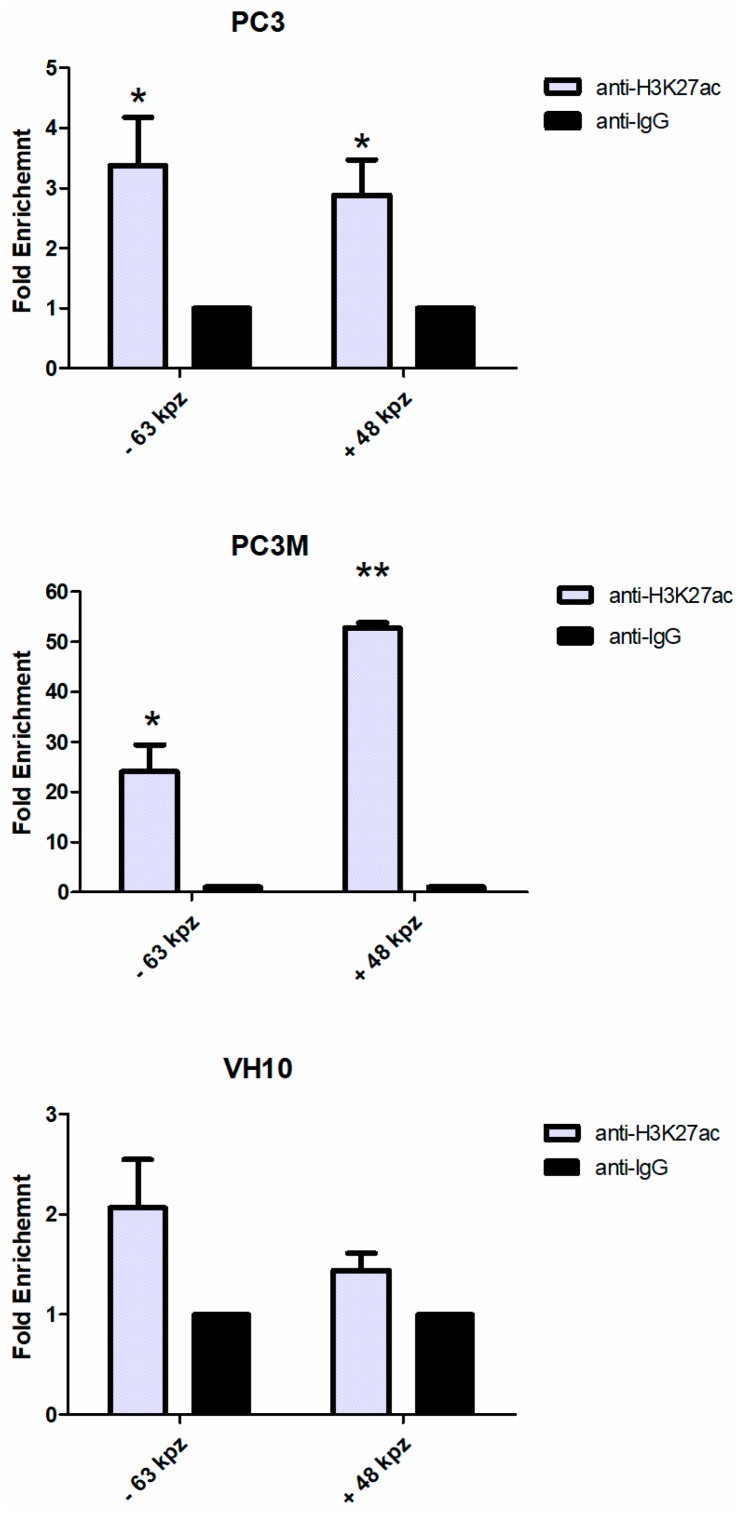
Histone acetylation across the *PTBP3* locus region in prostate cancer cells and skin fibroblasts. ChIP was performed using anti-H3K27Ac or a mouse control IgG antibody. H3K27Ac enrichment is shown relative to the IgG. Data are combined from at least two ChIP experiments. All data points were calculated as percentage of input material. Asterisks represent statistically significant changes in H3K27ac enrichment versus anti-IgG, with * *p* < 0.05 and ** *p* < 0.001.

In the PC3M cells, the fragment of interest located 48 kb downstream of the promoter region was significantly (over 60-fold) enriched in H3K27 acetylation. The second analyzed fragment located at −63 kb of the *PTBP3* locus was also significantly (over 30-fold) enriched in H3K27ac (Figure 3).

In the PC3 cells, an almost 5-fold and a 3-fold enrichment in H3K27Ac at the −63 kb and +48 kb positions, respectively, was found. The H3K27ac signal levels of fibroblast cell line (VH10) chromatin were not significantly different between the background signal obtained with an Immunoglobulin G (IgG) antibody across the analyzed regions of interests (−63 kb and +48 kb). In both prostate cancer cell lines, PC3M and PC3, ChIP assays targeting H3K27ac revealed that this type of histone modification was located at the same positions as those identified by the 3C assay of long-range chromatin interactions with the *PTBP3* promoter.

Results obtained in ChIP assay led us to *in silico* prediction of enhancer elements in the *PTBP3* locus. Our 3C approach employed the HindIII restriction enzyme and produced several DNA fragments of 1–16 kb in size. However, transcriptional enhancers are short DNA fragments (200–1500 bp) [32] that make it necessary to narrow down the likely position of the putative enhancers within the obtained −63 kb and +48 kb regions in the active *PTBP3* gene. To identify any putative enhancer elements localized in these upstream and downstream regions of *PTBP3*, respectively, we searched the Dragon ENhancers database (DENdb), which predicts enhancers derived from 15 human cell lines form the Encyclopedia of DNA elements (ENCODE) project [33]. DENdb determines enhancers using five different methods (ChromHMM [34], Segway [35], Random-Forest Based Algorithm for Enhancer Identification from Chromatin State RFECS [36], Chromatin signature identification by artificial neural network (CSI-ANN) [37] and ENCODE [38] integrated annotation).

In DENdb, we searched for putative enhancers focused on chromatin interacting positions of the active *PTBP3* gene: −63 kb (chr9: 115123248) and +48 kb (chr9: 115011604). We narrowed the −63 kb region down to two putative regions with enhancers: chr9:115123100–115123500 in lymphoblastoid cell line (Gm12878) and chr9: 115122550–115125100 in cervical cancer cell line (HeLa-S3). In the +48 kb region, we also found two putative enhancer regions in cancerous cell lines: at position chr9: 115010400–115013400 in the Gm12878 lymphoblastoid cell line, and at position chr9: 115010700–115012700 in the HeLa-S3 cervical cancer cell line.

## 3. Discussion

One of the epigenetic mechanisms of regulation of gene expression are chromatin conformation changes. Three-dimensional (3D) chromatin looping brings widely-separated distant gene regulatory elements into close spatial proximity with gene promoter to facilitate long-range control of gene expression [8,12,39]. Chromatin conformation capture (3C) provides information on the 3D chromatin organization of the analyzed loci that typically cover only ten to several hundred kb, using locus-specific primers. In this study, we used 3C to identify long-range chromatin interactions that may be mechanisms of the *PTBP3* gene expression regulation. We explored the spatial chromatin architecture of nearly 200 kb of chromosome 9 encompassing the *PTBP3* gene.

Previous results of studies of *PTBP3* expression indicated various expression patterns [20,28,29]. Our consistent qPCR and ddPCR results indicate a significantly increased level of *PTBP3* mRNA in prostate cancer cell lines compared to skin fibroblasts. To confirm that PTBP3 mRNA is overexpressed in prostate cancer cells, we evaluated the *PTBP3* expression pattern between prostate cancer and normal tissues samples [on the basis of Gene Expression Omnibus(GEO) and Gene Expression Profiling Interactive Analysis( GEPIA) annotations]. Microarray data obtained from GEO indicate that PTBP3 is upregulated in prostate tumors versus normal prostate tissue (GDS2545/35600; GDS4114/207223). Moreover, upregulation of the *PTBP3* gene has been demonstrated in prostate cancer progression states versus benign prostate cancer samples (GDS1439/224618). RNA sequencing expression data from the GEPIA analysis of the *PTBP3* expression across normal and cancerous prostate tissue also demonstrate upregulation of the *PTBP3* gene in prostate cancer tissue compared to normal samples (Figure S4, Supplementary data). However, the underlying genetic mechanisms responsible for conferring these different *PTBP3* expression patterns are still poorly understood.

We evaluated the level of the PTBP3 protein in the freely available Human Protein Atlas database (www.proteinatlas.org). As expected, based on the higher PTBP3 expression in prostate cancer cell lines, a high protein level was detected in many human cancers: lung, stomach, colorectal, urothelial and pancreatic (www.proteinatlas.org). Furthermore, overexpression of the PTBP3 protein in lung and pancreatic cancers was associated with unfavorable prognosis.

However, mechanisms which regulate the *PTBP3* expression in cancer cells have never been explored. Therefore, we examined cancerous and non-cancerous cell lines differing in the *PTBP3* mRNA levels to identify the spatial chromatin architecture of the *PTBP3* locus that can affect the activation of gene expression in *PTBP3*-expressing cells.

Our study revealed two elements (−63 kb and +48 kb) in a 200-kb region across the locus that looped specifically to the *PTBP3* promoter exclusively in *PTBP3*-expressing prostate cancer cell lines. As expected, no chromatin interactions were identified in the same region in non-cancerous skin fibroblasts with a low level of expression of the *PTBP3* gene.

We identified for the first time novel long-range chromatin interactions between the −63 kb and +48 kb loci and the *PTBP3* promotor which activate *PTBP3* expression in the cancerous prostate cell lines: PC3 and PC3M. In normal skin fibroblasts, flexible chromatin fibers are observed, with absence of specific long-range looping interactions in the *PTBP3* locus. These data suggest that in skin fibroblasts, there is no interaction within the region spanning 200 kb which could bring distal regulatory elements close to the gene promoter.

Enhancer elements are known to be associated with certain histone modifications—acetylation of lysine 27 in histone H3 (H3K27ac) and methylation of H3K4 [40]. H3K27ac is an important marker for enhancer elements that allows distinguishing between active and inactive enhancer regions according to the expression of proximal genes, in contrast to H3K4me1 that occurs in all enhancers [31]. Moreover, changes in the modifications of active histones, such as histone H3 acetylation, are also important in the three-dimensional organization of chromatin [41]. Therefore, we evaluated chromatin H3K27 acetylation at looping positions across the *PTBP3* gene. Chromatin immunoprecipitation was carried out in the prostate cancer cell lines PC3 and PC3M, and skin fibroblasts VH10 using antibodies specific for acetylated H3K27.

In the PC3 and PC3M cell lines with a highly active *PTBP3* locus, H3K27ac enrichment was detected at the analyzed −63 kb and +48 kb from the transcription startsites. In skin fibroblasts, in which the *PTBP3* locus is inactive, no statistical evidence for acetylated H3K27 was obtained at the analyzed positions.

H3K27 acetylation detected at analyzed regions of the *PTBP3* locus in prostate cancer cells (PC3 and PC3M) may suggest that open chromatin regions are located at positions −63 kb and +48 kb of the *PTBP3* promoter, respectively, and may act as epigenetic activation regulators of the *PTBP3* gene.

Data from DENdb, compared to our 3C and ChIP assay results, suggest a potential presence of distal enhancer elements that come into physical contact with proximal promoter via looped out chromatin in the prostate cancer cell lines PC3 and PC3M. The long-range chromatin looping interactions obtained in our study correlated with the overexpression of the *PTBP3* gene in prostate cancer cell lines.

Using the 3C assay and its derivative methods, it has previously been demonstrated that long-range chromatin interactions regulate many well-known genes associated with prostate cancer through chromatin looping. The abnormal activation of the androgen receptor (AR) is a major example of chromatin looping mechanisms that enhance gene expression in prostate cancer [42]. Recent studies have demonstrated that distal AR binding sites regulate well-known AR target genes, such as *PSA* Prostate specific antigen), *TMPRSS2* (Transmembrane serine protease 2), *UGT1A* (UDP glucuronosyltransferase family 1 member A complex locus), *UBE2C* (Ubiquitin conjugating enzyme E2 C), through chromatin looping [43,44,45,46]. Moreover, the chromosomal loops of *UBE2C* have been reported to be a new therapeutic target in castration-resistant prostate cancer [41,46].

**Figure 4:**
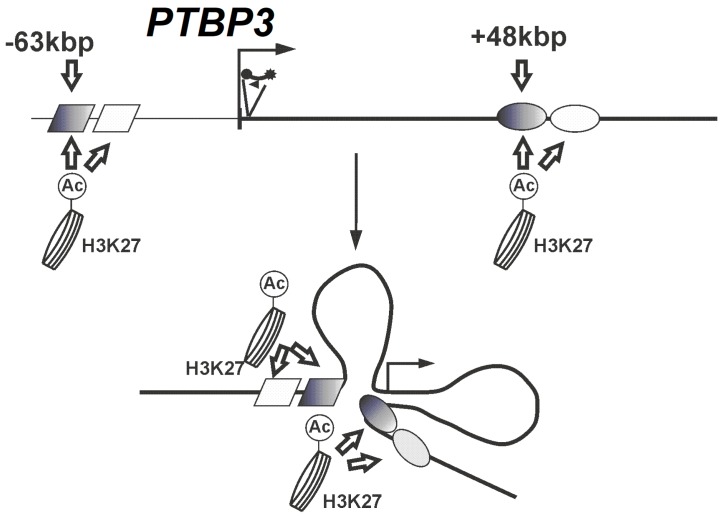
Looping model for *PTBP3* gene in prostate cancer cell lines. The bent arrow represents transcriptional start site of the *ROD1(PTBP3)* gene; the black circle with a star represent 3C Taqman probe (bait). The ellipses and diamonds represent the potential regulatory elements: grey diamond represents the region of chr9: 115123100–115123500 (DENdb); white diamond represents the region of chr9: 115122550–115125100 (DENdb); grey ellipse represents the region of chr9: 115010400–115013400 (DENdb); white ellipse represents the region of chr9:115010700–115012700 (DENdb).

Our results are consistent with the present of three-dimensional structural model and open chromatin form of the *PTBP3* gene in prostate cancer cell lines (Figure 4). This proposed looping model is clearly correlated with the expression of *PTBP3* and suggests that chromatin conformation changes are the main epigenetic mechanism that regulates *PTBP3* expression in prostate cancer cells.

## 4. Materials and Methods

### 4.1. Cell Line Cultures

The human prostate cancer cell lines PC3 and PC3M were grown in the Roswell Park Memorial Institute (RPMI) 1640 medium supplemented with 10% fetal bovine serum (FBS), and the human skin fibroblast cell line (VH10) was grown in Dulbecco’s Modified Eagle’s Medium (DMEM) supplemented with 10% Fetal Bovine Serum (FBS). The tested cell lines were cultured at 37 °C in a humidified atmosphere of 5% CO_2_. Cells were passaged every 2–3 days for exponential growth.

### 4.2. RNA Isolation and cDNA Synthesis

The total RNA from human prostate cancer cell lines (PC3, PC3M) and fibroblasts (VH10) was isolated using the High Pure RNA Isolation Kit (Roche, Mannheim, Germany) according to the manufacturer’s protocol. Complementary DNA (cDNA) was generated used the Transcriptor High Fidelity cDNA Synthesis Kit (Roche, Mannheim, Germany) with Anchored-oligo(dT)18 Primer. Denaturation of the template–primer mixture was carried out by heating the tube for 10 min at 65 °C. Reverse transcription was carried out at 50 °C for 30 min and at 85 °C for 5 min. The purity of cDNA was tested with agarose gel electrophoresis.

### 4.3. Real-time Reverse Transcriptase Polymerase Chain Reaction (RT-PCR)

Quantitative reverse transcriptase real-time PCR (qRT-PCR) was performed using RealTime Ready Custom Panels 96-8 (Roche, Mannheim, Germany) with primers and probe from Universal ProbeLibrary (Roche, Mannheim, Germany) specific to the *PTBP3*, *GAPDH* and *PBGD* genes, and run with LC480 Probes Master (Roche, Mannheim, Germany). The experiment was performed in triplicates for each cell line. Real-time PCR cycling conditions were as follows: One cycle at 95 °C, 10 min; 45 cycles of denaturation (95 °C, 10 s), annealing (60 °C, 30 s) extension (72 °C, 1 s) and cooling (4 °C, ∞).

### 4.4. Digital Droplet Polymerase Chain Reaction (ddPCR)

Digital droplet PCR was conducted to quantify the concentration of the *R* transcripts in prostate cancer cells and skin fibroblasts. TaqMan Gene Expression Assay was used to confirm the *PTBP3* gene expression profile by another quantifying method: ddPCR using the QX100 system (Bio-Rad, Hercules, CA, USA). The copy numbers for each target were averaged across duplicates and normalized to PBGD references genes. Endogenous mRNA levels were measured by ddPCR using the QuantaLife droplet digital PCR system (Bio-Rad, Hercules, CA, USA) according to the manufacturer’s instructions. Briefly, the 20-μL ddPCR mixture was prepared by combining cDNA, primers and probes with ddPCR Supermix (Bio-Rad, Hercules, CA, USA). Oil emulsion droplets for each sample were prepared using the QuantaLife droplet generator (Bio-Rad, Hercules, CA, USA). PCR reaction was under the following conditions: One cycle at 95 °C for 10 min, 45 cycles of 95 °C for 30 s, 60 °C for 1 min, and 98 °C for 10 min in a standard thermal cycler (Bio-Rad, Hercules, CA, USA). Plates were read on a QuantaLife droplet reader (Bio-Rad, Hercules, CA, USA), and the concentrations (copy numbers) of the targets in the samples were determined using the QuantaSoft software (QuantaSoft Analysis Pro software version 1.0).

### 4.5. Gene Expression Databases

The datasets for the analysis and visualization of the PBP3 expression level in prostate tumor versus normal prostate samples were obtained from several gene expression repositories: Gene Expression Ominbus (GEO) [47], the Cancer Genome Atlas (TCGA) [48] and Genotype-Tissue Expression (GTEx) [49]. Additionally, the Gene Expression Profiling Interactive Analysis (GEPIA) web-based tool was used for comprehensive expression analyses based on TCGA and GTEx data [50].

### 4.6. Chromosome Conformation Capture (3C)

3C experiments were completed as described previously [51] with minor modifications. Briefly, up to 10 million exponentially growing cells were fixed with 1% formaldehyde for 10 min at room temperature. The cells were lysed in 5 mL of cold lysis buffer (10 mM Tris pH 8, 10 mM NaCl, 0.2% NP-40) including 1× protease inhibitor, and nuclei were collected by centrifugation. The resulting cell nuclei were pelleted in 0.5 mL of the appropriate 1.2× restriction buffer, and sodium dodecyl sulfate (SDS) was added to each tube to a final concentration of 0.3%. Following extraction with 1.8% Triton X-100, chromatin was digested overnight at 37 °C with 2000 U of HindIII (Roche, Mannheim, Germany). Ligations were performed in a total reaction volume of 6.5 mL of ligation buffer, using 100 U of T4 DNA ligase and incubation at 14 °C for 4 h, followed by further incubation at room temperature for 30 min. DNA was extracted with phenol–chloroform followed by ethanol precipitation. The efficacy of restriction enzyme digestion was examined using SYBR green qPCR analysis with specific primer sets (Supplementary data Table S3) and was found to be more than 85% for each 3C library. The concentrations of 3C libraries were determined by quantitative PCR using the concINT9R2 F/R primer set and compared to a genomic DNA reference of known concentration. Samples were subsequently diluted to a concentration of 100 ng/mL. The Taqman qPCR technique was used to quantify the ligation frequency of the *PTBP3* gene. The Taqman probe (bait) and the reverse primer were generated within a restriction fragment at the promoter, and multiple forward primers were designed within restriction sites in distal regions across the *PTBP3* locus spanning a genomic distance of 203,723 kb (Supplementary data Table S1). The visual representation of DNase-seq and ChIP-seq (ENCODE Project, 2012) freely available at Genome Browser (University of California, Santa Cruz, CA, USA; http://genome.ucsc.edu/encode/) was used to evaluate DNase I-hypersensitive sites in the *PTBP3* locus and the flanking region upstream and downstream. Multiple forward primers at −79.7 kb, −63.2 kb, −30.7 kb, −6.8 kb, +48.4 kb, +71.3 kb and +119.3 kb in reference to the *PTBP3* transcriptional start site were designed. To analyze ligation products across the 3C templates, 200 ng of a 3C template was used per 20 μL of Taqman qPCR reaction. To account for differences in independently prepared 3C samples, the qPCR data for each sample were normalized to the results obtained for primers designed for the housekeeping gene *ERCC3* (ERCC Excision Repair 3), located in an area with the same chromatin structure in all types of cells. Each qPCR reaction was performed in triplicate, and the data presented were the average of two to four independent experimental results for all PCR reactions.

### 4.7. Chromatin Immunoprecipitation (ChIP)

ChIP assays were performed using Magna ChIP™ G (Millipore, Burlington, MA, USA) protocol with minor modifications. Approximately 4–5 × 10^6^ cells (on a 100 mm culture dish) were cross-linked with 1% formaldehyde for 10 min at room temperature. The reaction was stopped with glycine at 0.125 M. The cells were washed with cold Phosphate buffered saline (PBS), scraped and collected by centrifugation. Nuclei preparation and chromatin digestion was carried out using SimpleChIP® Cell Lysis Buffers A & B and MNase from Cell Signaling Technology (2316 WZ Leiden, The Netherlands). Chromatin was digested with 1000 units of MNase at 37 °C for 30 min, followed by sonication to an average size of 150 bp to 500 bp. Chromatin equivalents of ~1 × 10^6^ cells were suspended in Dilution Buffer with 1 × protease inhibitor cocktail (Millipore, Burlington, MA, USA) and precleared by incubation with 5 µL of protein G magnetic beads for 1 h at 4 °C. An aliquot of chromatin was removed and saved as “Input”. The reminder of supernatant containing precleared chromatin was immunoprecipitated using antibodies: 2 µL of H3K27Ac or 1 µL of normal mouse IgG (17-683, Millipore, Burlington, MA, USA), 20 µL of protein G magnetic beads blocked with bovine serum albumin (BSA) (washed 2× with 100 µL of BSA 0.5%, 400 µL of Dilution Buffer, 20 µL of 25× protease inhibitor cocktail (PIC) and 50 µL of BSA 0.5%. Immunoprecipitations were performed overnight at 4 °C. Protein G bead–antibody/chromatin complexes were washed as described in the Magna ChIP™ G (Millipore, Burlington, MA, USA) protocol. Protein/DNA complexes were eluted, and cross-links were reversed by incubation with proteinase K (10 µg/mL) and Elution Buffer (Millipore, Burlington, MA, USA) for 2 h at 62 °C and 10 min at 95 °C. DNA was purified using spin columns as described in the Magna ChIP™ G (Millipore, Burlington, MA, USA) protocol. Enrichment was analyzed using SYBR Green qPCR. qPCR primers (Supplementary data Table S2) were located at the selected positions: −63 kb and +48 kb in the *PTBP3* gene.

## 5. Conclusions

Our study revealed two elements (−63 kb and +48 kb) in a 200-kb region across the locus that looped specifically to the *PTBP3* promoter exclusively in *PTBP3*-expressing prostate cancer cell lines. The model of three dimensional chromatin conformation in the PTBP3 locus obtained for prostate cancer cell lines with high PTBP3 expression suggests, that the variable chromatin conformation is a mechanism that regulates its expression.

## Supplementary Materials

Supplementary Materials can be found at http://www.mdpi.com/1422-0067/20/2/242/s1.
