# Supplementary figures and images for: Chromosome Conformation Capture Reveals Two Elements That Interact with the PTBP3 (ROD1) Transcription Start Site
Source: Int J Mol Sci. 2019 Jan 9;20(2):242. doi: 10.3390/ijms20020242 (PMC6359592; doi:10.3390/ijms20020242)

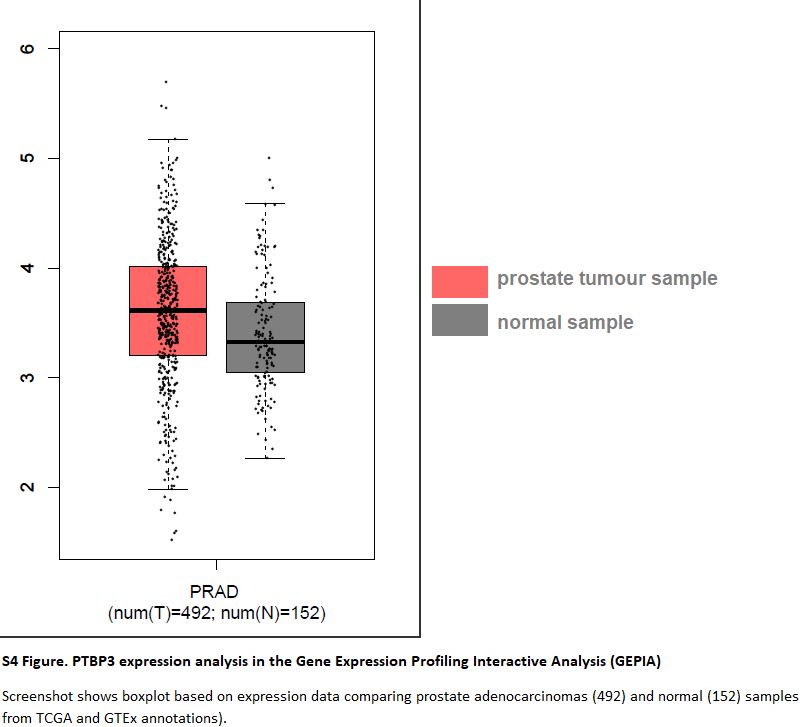

Supplement: Supplementary file 1 [file ijms-20-00242-s001.zip › S4 Figure-final.bmp]

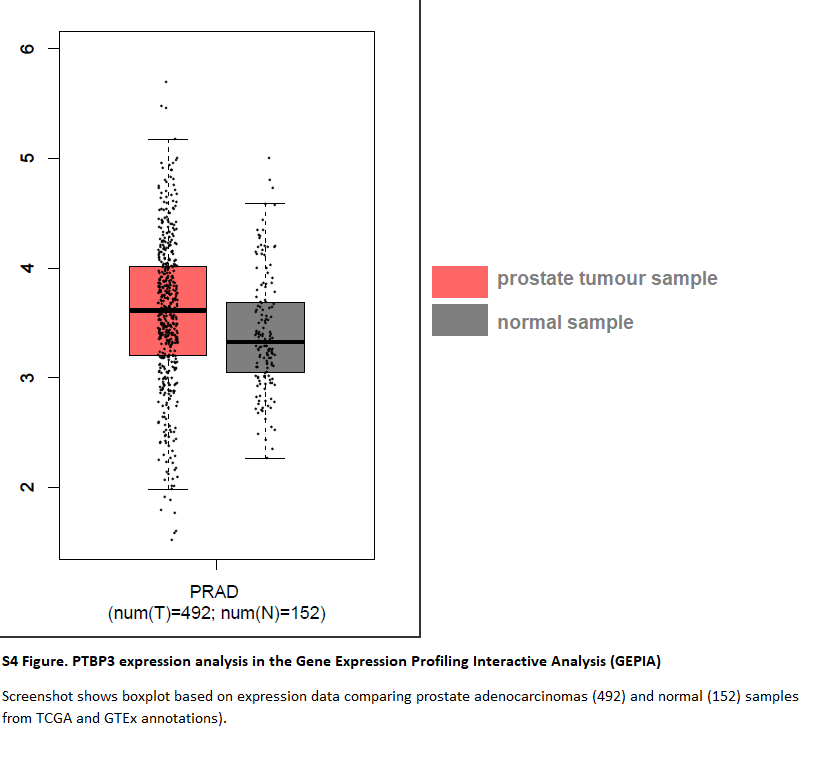

Supplement: Supplementary file 1 [file ijms-20-00242-s001.zip › Supplementary data word-final.docx]
